# Organizational stressors and athlete burnout in elite soccer players: the mediating role of basic psychological need frustration and the moderating role of coach autonomy support

**DOI:** 10.3389/fpsyg.2026.1933382

**Published:** 2026-09-14

**Authors:** Song Yuan, Yunmei Chai, Zhibo Cui, Quanhong Lu

**Affiliations:** 1 Football Academy, Chengdu Sport University, Chengdu, China; 2 School of Physical Education, Chengdu Sport University, Chengdu, China; 3 School of Sports Training, Tianjin University of Sport, Tianjin, China; 4 School of Sports Training, Chengdu Sport University, Chengdu, China

**Keywords:** athlete burnout, basic psychological need frustration, coach autonomy support, elite soccer players, moderated mediation model, organizational stressors

## Abstract

**Introduction:**

Athlete burnout is an important psychological state that may affect sustained training engagement, athletic performance, and mental health among elite soccer players. Drawing on basic psychological need theory, this study examined the association between organizational stressors and athlete burnout among Chinese elite soccer players with dual educational and sport-training roles, and tested whether basic psychological need frustration accounted for an indirect association and whether coach autonomy support served as a boundary condition.

**Methods:**

A cross-sectional online survey was conducted with 513 Chinese elite soccer student-athletes enrolled in the Football College of Chengdu Sport University. According to the college admission requirements and self-reported athletic-level information, all participants had attained at least the Chinese National First-Class Athlete level before or at university entry. Participants completed measures of organizational stressors, basic psychological need frustration, coach autonomy support, and athlete burnout. Data were analyzed using confirmatory factor analysis, correlation analysis, bootstrap tests of indirect associations, and a first-stage moderated mediation model.

**Results:**

Organizational stressors were positively associated with both athlete burnout and basic psychological need frustration. Basic psychological need frustration was positively associated with athlete burnout and accounted for a significant indirect association between organizational stressors and athlete burnout. Coach autonomy support showed a small but significant buffering effect on the association between organizational stressors and basic psychological need frustration, and the conditional indirect association through basic psychological need frustration was weaker at higher levels of coach autonomy support.

**Discussion:**

The findings suggest that athlete burnout among elite soccer players with dual educational and sport-training roles is closely associated with organizational stressors, and that basic psychological need frustration may help explain this statistical association. Coach autonomy support may constitute a contextual boundary condition that modestly reduces the association between organizational stressors and basic psychological need frustration. These results suggest that management of organizational stressors, basic psychological need experiences, and autonomy-supportive coaching behaviors may be relevant targets for future longitudinal and intervention research. Given the cross-sectional and self-reported nature of the data, the findings should be interpreted as theoretically specified statistical associations rather than evidence of strict causality.

## 1 Introduction

Elite soccer players are routinely exposed to high training loads, sustained competition, public performance evaluation, and uncertainty about career development. In the Chinese higher-education context, some soccer student-athletes enrolled in specialized sport colleges have already attained high competitive qualifications before or at university entry and continue to receive systematic soccer-specific training within a university-based training and education context. These athletes therefore occupy dual educational and sport-training roles. High-level athletic experience does not protect athletes from mental health difficulties. Injury, performance pressure, career transitions, and insufficient support may all increase the risk of poor psychological adjustment (Rice et al., 2016). Evidence from soccer also indicates that depression and anxiety are present among top-level male and female players (Junge and Feddermann-Demont, 2016). Therefore, attention to the psychological functioning of elite soccer players with dual educational and sport-training roles is important not only for individual wellbeing but also for training engagement, athletic performance, academic adjustment, and career sustainability.

The core outcome variable in this study is athlete burnout. Athlete burnout is generally understood as a chronic maladaptive psychological state that develops in the context of prolonged training demands, competitive pressure, and sport participation. It mainly comprises emotional and physical exhaustion, a reduced sense of accomplishment, and sport devaluation. Unlike temporary training fatigue or short-term post-competition exhaustion, athlete burnout reflects a more persistent depletion response to sustained stress exposure in sport (Gustafsson et al., 2011). On this basis, the present study treats athlete burnout as an important outcome of psychological adjustment associated with organizational stressors, and focuses on how organizational stressors may be linked to burnout through basic psychological need frustration.

Previous research has documented associations among organizational stressors, basic psychological need experiences, and athlete burnout (Pacewicz et al., 2019). However, in elite soccer contexts, further work is needed to examine whether organizational stressors are statistically associated with athlete burnout through more proximal need-related experiences, and which coaching conditions may buffer this association (Bartholomew et al., 2011a; Tabei et al., 2012; Wagstaff et al., 2018). This issue is particularly relevant for Chinese elite soccer players with dual educational and sport-training roles, because their organizational stressors may arise from overlapping educational and sport-performance systems. Basic psychological need theory proposes that autonomy, competence, and relatedness are essential for psychological integration and healthy functioning. When these needs are obstructed, individuals are more likely to experience maladaptive outcomes. Accordingly, this study examines elite soccer players with dual educational and sport-training roles and tests a theory-guided indirect association linking organizational stressors, basic psychological need frustration, and athlete burnout. It further investigates whether coach autonomy support moderates the association between organizational stressors and basic psychological need frustration.

## 2 Literature review and hypothesis development

### 2.1 Organizational stressors and athlete burnout

Organizational stressors often have a sustained and depleting effect on athletes’ psychological functioning (Fletcher et al., 2012). Organizational demands do not necessarily lead to negative outcomes. Clear training goals, reasonable selection rules, and appropriate performance requirements may provide athletes with a sense of direction. However, when coaching conflicts, role ambiguity, selection uncertainty, logistical problems, tense team climates, or limited developmental opportunities occur repeatedly, athletes must continually invest attentional, emotional, and self-regulatory resources to cope with these demands (Arnold and Fletcher, 2012). Prolonged exposure to such organizational conditions may gradually deplete athletes’ psychological resources and restrict recovery opportunities, thereby increasing the likelihood of burnout (Goodger et al., 2007).

In elite soccer, the association between organizational stressors and athlete burnout deserves particular attention. Soccer is highly dependent on teamwork, coaching decisions, and role allocation. Playing opportunities, field positions, team roles, and career prospects are often shaped by selection systems, coaches’ evaluations, and team culture (Brown and Potrac, 2009). When these organizational demands lack transparency, stability, or support, athletes must cope not only with training and competition loads, but also with uncertainty, perceived unfairness, and interpersonal pressure. These experiences may increase psychological energy expenditure and make athletes more vulnerable to difficulties in maintaining attention, emotional exhaustion, reduced willingness to train, and insufficient subjective recovery, all of which are closely related to burnout symptoms (Tabei et al., 2012; Wagstaff et al., 2018). Accordingly, this study proposes the following hypothesis:

*H1*: Organizational stressors will be positively associated with athlete burnout.

### 2.2 The mediating role of basic psychological need frustration

Basic psychological need theory proposes that autonomy, competence, and relatedness are essential for psychological integration, motivational regulation, and healthy functioning. Autonomy refers to the experience that one’s behavior is volitional and personally endorsed; competence reflects the experience of being effective in task completion and continued development; and relatedness concerns a sense of acceptance, care, and connection with significant others (Vansteenkiste et al., 2020).

Need frustration is not simply a low level of need satisfaction. It refers to the active experience of being controlled, feeling incompetent, or being rejected by others. Such experiences are more directly associated with psychological vulnerability and maladaptive outcomes. Basic psychological need frustration therefore provides a proximal psychological explanation for how organizational stressors may be associated with athlete burnout.

In elite soccer, organizational stressors may induce need frustration by threatening athletes’ autonomy, competence, and relatedness. When coaching decisions lack explanation, training arrangements leave little room for participation, role allocation is unclear, or selection rules are unstable, athletes may experience organizational demands as externally controlling, thereby leading to autonomy frustration. Ambiguous evaluation criteria, limited developmental opportunities, and unclear links between effort and outcomes may weaken athletes’ sense of ability and progress, giving rise to competence frustration. Coaching conflicts, teammate exclusion, tense team climates, or insufficient communication may make athletes feel misunderstood, unaccepted, or marginalized, thereby contributing to relatedness frustration (Bartholomew et al., 2011b; Fletcher et al., 2012; Haberstock et al., 2024). Research in sport has also shown that controlling coaching environments more strongly predict athletes’ basic psychological need frustration, which is in turn closely related to impaired psychological functioning and maladaptive outcomes (Bartholomew et al., 2011a). Accordingly, this study proposes the following hypothesis:

*H2*: Organizational stressors will be positively associated with basic psychological need frustration.

Basic psychological need frustration represents a more proximal experience of impaired psychological functioning than external stress events. Persistent autonomy frustration may weaken athletes’ internal endorsement of training and competition. Competence frustration may lead athletes to repeatedly experience ineffectiveness and insufficient ability. Relatedness frustration may increase feelings of alienation within the team and impose additional emotional regulation demands. Athlete burnout consists of emotional and physical exhaustion, a reduced sense of accomplishment, and sport devaluation (Raedeke and Smith, 2001). These dimensions are theoretically consistent with the experiences of control, incompetence, and impaired relationships that arise from need frustration (Bartholomew et al., 2011a; Vansteenkiste and Ryan, 2013). Existing studies in athletes have also shown stable associations between basic psychological need-related experiences, athlete burnout, and other maladaptive outcomes (Lonsdale et al., 2009). Accordingly, this study proposes the following hypothesis:

*H3*: Basic psychological need frustration will be positively associated with athlete burnout.

Taken together, these associations suggest an explanatory pathway from organizational environments to need experiences and then to mental health outcomes. Organizational stressors, as relatively distal contextual demands, may first be associated with athletes’ experiences of autonomy, competence, and relatedness. Basic psychological need frustration, as a more proximal psychological mechanism, may then link organizational stressors to athlete burnout. Given the cross-sectional questionnaire design of this study, the proposed mediation should be understood as a theoretically specified statistical mediation effect rather than a strict temporal causal chain. Accordingly, this study proposes the following hypothesis:

*H4*: Basic psychological need frustration will account for a significant indirect association between organizational stressors and athlete burnout.

### 2.3 The moderating role of coach autonomy support and moderated mediation

Coach autonomy support is an important form of need-supportive social context within self-determination theory. It mainly refers to coaching behaviors through which coaches acknowledge athletes’ perspectives and feelings, provide appropriate choices, explain the rationale for task demands, and reduce threatening, pressuring, or controlling language (Mageau and Vallerand, 2003). In competitive sport, autonomy support does not mean abandoning rules or lowering training standards. Rather, under conditions of high demands and strong competition, it involves using explanation, structure, feedback, and limited choice to help athletes understand requirements, endorse goals, and retain a basic sense of psychological agency (Cheon et al., 2015; Reynders et al., 2019; Mossman et al., 2024). For elite soccer players, coaches are not only organizers of training tasks, but also key agents through whom team systems, selection rules, and organizational demands are communicated to athletes. Coaches’ communication and support may therefore shape how athletes interpret and experience organizational stressors.

From the perspective of basic psychological need theory, coach autonomy support may weaken the positive association between organizational stressors and basic psychological need frustration. When athletes face intensive training, role competition, selection uncertainty, or developmental pressure, coaches who explain the reasons behind decisions, acknowledge athletes’ stress experiences, and provide opportunities for expression and feedback may reduce the extent to which organizational demands are experienced as one-sided pressure (Isoard-Gautheur et al., 2012). Clear feedback can help athletes understand how to improve, while respectful communication may help maintain relational security. By contrast, when coach autonomy support is low, the same organizational stressors may be more readily interpreted as control, failure, or relational alienation, thereby intensifying basic psychological need frustration. Previous research has shown that perceived autonomy support is closely associated with basic need satisfaction and positive psychological functioning (Adie et al., 2008). Among elite youth soccer players, coach autonomy support, basic need satisfaction, and athletes’ well- and ill-being also show longitudinal associations (Adie et al., 2012). In combination with evidence on controlling sport environments and need frustration (Haberstock et al., 2024), this study proposes the following hypothesis:

*H5*: Coach autonomy support will moderate the association between organizational stressors and basic psychological need frustration, such that this association will be weaker at higher levels of coach autonomy support.

If basic psychological need frustration mediates the relationship between organizational stressors and athlete burnout, and coach autonomy support weakens the association between organizational stressors and basic psychological need frustration, then the indirect effect of organizational stressors on athlete burnout through basic psychological need frustration may also vary across levels of coach autonomy support. Specifically, when coach autonomy support is low, organizational stressors may be more strongly associated with athlete burnout through basic psychological need frustration. When coach autonomy support is high, this indirect association should be relatively weaker. Accordingly, this study proposes the following hypothesis:

H6: The indirect association between organizational stressors and athlete burnout through basic psychological need frustration will vary across levels of coach autonomy support.

## 3 Materials and methods

### 3.1 Participants and procedure

This study used a cross-sectional online questionnaire design and recruited Chinese elite soccer student-athletes enrolled in the Football College of Chengdu Sport University. In this study, elite soccer student-athletes were operationally defined as undergraduate or graduate students who had received systematic soccer-specific training and had attained at least the Chinese National First-Class Athlete level before or at university entry. According to the college admission requirements and self-reported athletic-level information, all participants met this criterion. The study focused on this specific group of university-based elite soccer student-athletes rather than on athletes recruited from multiple professional clubs, provincial teams, or external training organizations. Questionnaires were excluded if they showed missing values on core variables, clearly abnormal response times, patterned responses, or incomplete responses to the main measurement items.

The study was reviewed and approved by the Ethics Committee of the Football College, Chengdu Sport University on 8 June 2026 (approval no. [2026] 203), before data collection. Data were collected online from 9 June 2026 to 1 July 2026 through a coach group of the national top-level collegiate league. Before completing the survey, participants were informed about the purpose of the study, anonymity, confidentiality, data use, voluntary participation, and their right to withdraw at any time. Participants began the questionnaire only after providing informed consent. For participants younger than 18 years, informed consent was obtained from their legal guardians, and assent was obtained from the participants themselves. All participants were undergraduate or graduate students enrolled in the Football College of Chengdu Sport University. According to the admission requirements of the college and the self-reported athletic-level information, all participants had attained at least the Chinese National First-Class Athlete level before or at university entry. Participants came from different provincial-level regions of mainland China; this information refers to their places of origin rather than to recruitment from multiple teams, clubs, or training organizations. The survey did not record identifiable team, club, university-team, or training-unit information. A total of 532 questionnaires were initially returned. After excluding invalid responses with missing values on core variables (*n* = 3), clearly abnormal response times (*n* = 3), patterned responses (*n* = 11), and incomplete responses to the main measurement items (*n* = 2), 513 valid questionnaires were retained for analysis. The database was locked on 1 July 2026, and all data screening and statistical analyses reported in the originally submitted manuscript were completed before submission on 9 July 2026. All valid responses included complete data on the core variables and background variables and were included in the subsequent statistical analyses. The distributions of sex, age, academic year, athletic level, playing position, years of soccer-specific training, team role, and training load are shown in Table 1 and Supplementary Table 1. All data were de-identified before being used for academic research and statistical analysis.

**TABLE 1:** Demographic and training characteristics of the sample (*N* = 513).

Characteristic	Category	*n*	%	Characteristic	Category	*n*	%
Sex	Male	303	59.1	Age	Under 18 years	22	4.3
	Female	210	40.9		1–21 years	166	32.4
Academic year	First year	79	15.4		22–24 years	192	37.4
	Second year	109	21.2		25–27 years	94	18.3
	Third year	96	18.7		27 years or older	39	7.6
	Fourth year	96	18.7	Years of soccer-specific training	Less than 3 years	50	9.7
	Graduate student	133	25.9		3–5 years	169	32.9
Playing position	Goalkeeper	86	16.8		6–8 years	177	34.5
	Defender	94	18.3		9–11 years	87	17
	Midfielder	96	18.7		12 years or more	30	5.8
	Forward	123	24	Weekly soccer-specific training frequency	1–2 sessions	47	9.2
	Multiple positions	114	22.2		3–4 sessions	162	31.6
Team role	Starting player	229	44.6		5–6 sessions	202	39.4
	Main rotation player	145	28.3		7 sessions or more	102	19.9
	Substitute player	92	17.9	Duration of each training session	Less than 1 hour	37	7.2
	Temporarily not competing	47	9.2		1–2 hours	151	29.4
Athletic level	National First-Class Athlete	374	72.9		2–3 hours	233	45.4
	National Master Athlete	139	27.1		More than 3 hours	92	17.9

### 3.2 Measures

This study used four core measurement instruments to assess athlete burnout, coach autonomy support, basic psychological need frustration, and organizational stressors. All scales were rated on a 5-point Likert scale, with higher scores indicating higher levels of the corresponding construct. Because the participants were Chinese elite soccer players with dual educational and sport-training roles, the wording of the measurement items was reviewed to ensure contextual relevance to soccer training and competition. The results for internal consistency, composite reliability, convergent validity, and discriminant validity are reported in the Results section.

#### 3.2.1 Athlete burnout

Athlete burnout was measured using the Athlete Burnout Questionnaire (ABQ). The scale was developed by Raedeke and Smith to assess burnout symptoms among athletes in the context of sustained training, competitive pressure, and sport participation (Raedeke and Smith, 2001). In the present soccer training and competition context, the scale was used to assess athletes’ emotional and physical exhaustion, reduced sense of accomplishment, and sport devaluation. The scale includes 15 items covering three dimensions: emotional and physical exhaustion, reduced sense of accomplishment, and sport devaluation. Items AB1 and AB14 were reverse-coded according to the scoring instructions for the version used before data analysis. After reverse coding, item scores were summed to form the athlete burnout total score, with higher scores indicating higher levels of burnout. The Cronbach’s alpha for this scale was 0.957.

#### 3.2.2 Perceived coach autonomy support

Coach autonomy support was measured using the short form of the autonomy support scale developed by Williams et al., which has shown good internal consistency (Williams et al., 1996). Based on the self-determination theory definition of autonomy-supportive coaching behavior, the scale mainly assessed athletes’ perceptions of whether their coaches understood their perspectives, provided opportunities for choice, encouraged expression of opinions, and offered reasonable explanations when setting training or competition demands. The wording was reviewed to ensure that the referent of support was the coach in the soccer training and competition context. The scale includes six items. Item scores were summed to form the coach autonomy support total score, with higher scores indicating higher levels of perceived autonomy support. The Cronbach’s alpha for this scale was 0.904.

#### 3.2.3 Basic psychological need frustration

Basic psychological need frustration was measured using the Basic Psychological Need Satisfaction and Frustration Scale developed by Chen et al. (2015). The scale was used to assess the extent to which athletes’ needs for autonomy, competence, and relatedness were obstructed in soccer training and competition contexts (Bartholomew et al., 2011b). The scale includes 12 items covering three dimensions: autonomy frustration, competence frustration, and relatedness frustration. Item scores were summed to indicate the overall level of basic psychological need frustration, with higher scores indicating stronger need frustration. The Cronbach’s alpha for this scale was 0.949.

#### 3.2.4 Organizational stressors

Organizational stressors were measured using the scale developed by Arnold, Fletcher, and Daniels. This scale assesses organizational-level stress demands experienced by athletes over the previous 3 months, distinguishing these demands from immediate competitive stress during performance (Arnold et al., 2013). In the present study, organizational stressors referred to stressors arising from the soccer training and competition environment, including coaching, team culture, logistics, selection, and goals and development. The scale includes 21 items covering these five dimensions. Item scores were summed to form the organizational stressors total score, with higher scores indicating higher levels of perceived organizational stressors. The Cronbach’s alpha for this scale was 0.971.

#### 3.2.5 Background variables

The present study recorded background variables including sex, age, academic year, athletic level, playing position, years of soccer-specific training, team role, weekly frequency of soccer-specific training, and duration of each training session. These variables were used to describe the sample characteristics and to define the scope within which the findings may be applicable. Specifically, sex was used to describe the basic demographic composition of the sample; age and academic year reflected the athletes’ developmental and educational stages; athletic level was used to indicate competitive level; and playing position, years of soccer-specific training, team role, weekly training frequency, and training-session duration were used to describe athletes’ soccer-specific experience, participation status within the team, and training exposure.

In the statistical analyses, background variables were coded according to the response categories used in the questionnaire. Sex and athletic level were treated as binary variables; age, academic year, years of soccer-specific training, weekly training frequency, and training-session duration were organized according to the ordered categories specified in the questionnaire; and playing position and team role were treated as categorical variables. In the regression analyses, categorical covariates were dummy-coded rather than selected on the basis of univariate statistical significance. In addition to reporting the unadjusted model, the present study also established prespecified covariate-adjusted models. The main adjusted model simultaneously included sex, age group, athletic level, team role, years of soccer-specific training, weekly frequency of soccer-specific training, and training-session duration in both the mediator equation and the outcome equation. The extended adjusted model further included academic year and playing position, and used ordinal trend coding for age categories as a sensitivity analysis. By reporting both unadjusted and covariate-adjusted models, this study examined whether the core associations among organizational stressors, basic psychological need frustration, coach autonomy support, and athlete burnout remained consistent after controlling for relevant demographic, competitive-experience, and training-related characteristics.

### 3.3 Statistical analysis

Data were analyzed using R and SPSS PROCESS 5.0. Confirmatory factor analysis, reliability and validity testing, descriptive statistics, and correlation analyses were conducted in R. Indirect effects, moderation effects, and the first-stage moderated mediation effect were tested using PROCESS Model 7, with 95% confidence intervals estimated based on 5,000 bootstrap samples. Before the formal analyses, questionnaires were screened for completeness, missing values on core variables, response time, patterned responses, and demographic and training-related information. Questionnaires with missing data on core variables, clearly invalid responses, or extremely abnormal response patterns were excluded. Before regression and interaction analyses, the distributional characteristics of continuous variables were examined, and mean-centering was performed as required by the model.

Confirmatory factor analysis was primarily conducted using the weighted least squares mean- and variance-adjusted estimator (WLSMV). Based on the theoretical structure of each scale, the three-factor model of athlete burnout, the three-factor model of basic psychological need frustration, the five-factor model of organizational stressors, and the one-factor model of coach autonomy support were tested separately. Correlated first-order factor models and higher-order factor models were further estimated. On this basis, the discriminant validity of the four latent constructs, namely organizational stressors, basic psychological need frustration, coach autonomy support, and athlete burnout, was examined by comparing a 12-factor model, a second-order factor model, a four-factor model, and a single-factor model. For applicable models, robust maximum likelihood estimation (MLR) was used for sensitivity analyses to examine whether the conclusions regarding model fit and parameter admissibility were consistent across estimators. Model fit was evaluated using χ^2^/df, RMSEA, SRMR, CFI, and TLI (Hu and Bentler, 1999; Kline, 2023). When χ^2^/df was below 3, RMSEA and SRMR were below 0.08, and CFI and TLI were above 0.90, model fit was considered acceptable. Internal consistency reliability was assessed using Cronbach’s alpha and composite reliability (CR), and convergent validity was examined using standardized factor loadings and average variance extracted (AVE) (Cronbach, 1951; Fornell and Larcker, 1981). Cronbach’s alpha and CR values above 0.70 and AVE values above 0.50 were considered to indicate acceptable internal consistency reliability and convergent validity.

Because all variables were collected at the same time point using self-report questionnaires, Harman’s single-factor test was used as a preliminary assessment of common method bias (Podsakoff et al., 2003). All measurement items for the core variables were entered into an unrotated exploratory factor analysis, and the proportion of total variance explained by the first factor was examined. Because this method cannot fully rule out same-source measurement effects, the findings should be interpreted in light of the theoretical model, measurement model, statistical results, and the limitations of cross-sectional self-report data.

Descriptive statistics included means, standard deviations, skewness, and kurtosis, and Pearson correlation coefficients were used to examine bivariate relationships among the main variables. The associations between organizational stressors and athlete burnout, between organizational stressors and basic psychological need frustration, and between basic psychological need frustration and athlete burnout were used to test H1, H2, and H3, respectively. Indirect effects and conditional indirect effects were estimated using 5,000 bootstrap samples, and effects were considered significant when the 95% confidence interval did not include zero (Preacher and Hayes, 2008).

The moderation effect was tested using the interaction term between organizational stressors and coach autonomy support. When the interaction term was significant, simple slope analyses were further conducted to examine the association between organizational stressors and basic psychological need frustration at low, mean, and high levels of coach autonomy support, defined as one standard deviation below the mean, the mean, and one standard deviation above the mean, respectively (Aiken, 1991). When necessary, the Johnson–Neyman method was used to identify regions of significance (Johnson and Neyman, 1936; Hayes and Matthes, 2009). Finally, the moderated mediation effect was examined using conditional indirect effects and the index of moderated mediation. H6 was considered supported when the bootstrap 95% confidence interval did not include zero (Hayes, 2015).

## 4 Results

### 4.1 Participant characteristics and data screening

A total of 513 valid questionnaires were included in the study. All participants were soccer players with relatively high competitive levels, systematic soccer-specific training experience, and dual educational and sport-training roles. The demographic and training characteristics of the sample are presented in Table 1. The sample included 303 male athletes (59.1%) and 210 female athletes (40.9%). Most participants were between 19 and 24 years of age, including 166 athletes aged 19–21 years (32.4%) and 192 athletes aged 22–24 years (37.4%). In terms of athletic level, 374 participants were Chinese National First-Class Athletes (72.9%) and 139 were National Master Athletes (27.1%), which was consistent with the operational definition of elite soccer players used in this study.

The sample covered different playing positions, team roles, and training load levels. Regarding playing position, 123 participants were forwards (24.0%), 114 played multiple positions (22.2%), 96 were midfielders (18.7%), 94 were defenders (18.3%), and 86 were goalkeepers (16.8%). In terms of team role, 229 participants were starting players (44.6%), 145 were main rotation players (28.3%), 92 were substitute players (17.9%), and 47 were temporarily not competing (9.2%). The most common duration of soccer-specific training was 6–8 years, reported by 177 participants (34.5%). The most common weekly soccer-specific training frequency was 5–6 sessions, reported by 202 participants (39.4%). The most common duration of each training session was 2–3 h, reported by 233 participants (45.4%).

Data screening showed that all 513 questionnaires had complete responses for the nine background variables and 54 psychological measurement items, and all were included in the subsequent statistical analyses. The absolute skewness values for organizational stressors, basic psychological need frustration, coach autonomy support, and athlete burnout ranged from 0.017 to 0.068, and the absolute kurtosis values ranged from 0.969 to 1.130. These results did not indicate substantial deviations from normality.

### 4.2 Measurement model, reliability, validity, and common method bias

Because all variables in this study were measured using self-report questionnaires at the same time point, potential common method bias (CMB) may have influenced the statistical associations among the variables. Therefore, Harman’s single-factor test was first used to provide a preliminary assessment of common method bias. The results showed that the first principal component extracted from the unrotated principal component analysis explained 37.75% of the total variance, which was below the reference criterion of 40%. Thus, no single factor was found to account for the majority of the variance.

As shown in Table 2, all study variables demonstrated good internal consistency and convergent validity. Cronbach’s alpha coefficients for the scales ranged from 0.904 to 0.971, all exceeding the recommended threshold of 0.70, indicating high internal consistency. The standardized factor loadings of the measurement items ranged from 0.766 to 0.859, all above 0.70, suggesting that the items adequately reflected their corresponding latent variables. In addition, the composite reliability (CR) values of the constructs ranged from 0.925 to 0.978, all exceeding the recommended threshold of 0.70. The average variance extracted (AVE) values ranged from 0.658 to 0.675, all above the criterion of 0.50, further supporting the good convergent validity of the broad constructs.

**TABLE 2:** Reliability and convergent validity of the scales.

Construct	Items	*M*	SD	α	Standardized factor loading range	CR	AVE
Organizational stressors	21	69.528	20.016	0.971	0.798–0.859	0.978	0.675
Basic psychological need frustration	12	40.004	11.224	0.949	0.766–0.842	0.960	0.668
Coach autonomy support	6	15.947	5.866	0.904	0.802–0.853	0.925	0.674
Athlete burnout	15	49.676	13.878	0.957	0.782–0.837	0.966	0.658

As shown in Table 3, although the 12-factor model and the second-order factor model estimated using WLSMV both showed excellent overall model fit, the factor correlations among the internal dimensions of the ABQ, BPNF, and OSI were close to or exceeded 1. In addition, problems such as a non-positive definite latent variable covariance matrix, standardized second-order factor loadings reaching or exceeding 1, and negative residual variances were observed. These results indicate that, although the above models showed favorable global fit indices, their parameter estimates were statistically inadmissible. Therefore, these models did not support sufficient empirical distinctiveness among the theoretical subdimensions of the scales in the present sample.

**TABLE 3:** Measurement-model comparisons.

Model	Estimator	χ ^2^	df	χ ^2^/df	CFI	TLI	RMSEA	SRMR
Twelve-factor model	WLSMV	1257.2	1,311	0.959	1	1	0.000	0.026
Second-order factor model	WLSMV	1306.5	1,360	0.961	1	1.001	0.000	0.028
Four-factor model	WLSMV	1313.8	1,371	0.958	1	1	0.000	0.028
One-factor model	WLSMV	10791.2	1,377	7.837	0.821	0.814	0.116	0.158
Second-order factor model	MLR	1442	1,360	1.06	0.996	0.996	0.011	0.027
Four-factor model	MLR	1452.4	1,371	1.059	0.996	0.996	0.011	0.027
One-factor model	MLR	9768.9	1,377	7.094	0.564	0.547	0.109	0.145

By contrast, the four-factor model, consisting of OSI, BPNF, CAS, and AB, showed good model fit and acceptable parameter estimates under both WLSMV and MLR estimation. Specifically, the MLR results were χ^2^ (1,371) = 1452.40, CFI = 0.996, TLI = 0.996, RMSEA = 0.011, and SRMR = 0.027. The single-factor model showed poor fit under both estimation methods. Further analyses indicated good discriminant validity among the four broad constructs. The absolute values of the latent variable correlations and the HTMT values ranged from 0.426 to 0.497, and the square roots of AVE for the constructs, ranging from 0.811 to 0.822, were all higher than the corresponding latent variable correlations.

Therefore, although the scale items demonstrated good reliability and convergent validity at the level of broad constructs, some theoretical subdimensions did not show sufficient empirical distinctiveness in the present sample. Based on these measurement model results, the subsequent structural model analyses were conducted using the four broad constructs of OSI, BPNF, CAS, and AB, rather than independently interpreting the internal subdimensions of each scale.

### 4.3 Descriptive statistics, correlation analysis, and discriminant validity

The results are presented in Table 4 and Figure 1. Correlation analyses showed that OSI was significantly and positively correlated with BPNF (*r* = 0.410, *p* < 0.001) and AB (*r* = 0.451, *p* < 0.001), and significantly and negatively correlated with CAS (*r* = −0.418, p < 0.001). BPNF was significantly and positively correlated with AB (*r* = 0.385, *p* < 0.001), and significantly and negatively correlated with CAS (*r* = −0.369, *p* < 0.001). CAS was significantly and negatively correlated with AB (*r* = −0.386, *p* < 0.001). The directions of the correlations among the variables were consistent with theoretical expectations. Regarding discriminant validity, the square root of AVE for each variable was greater than its correlations with the other variables, indicating good discriminant validity among the constructs.

**TABLE 4:** Descriptive statistics, correlations, and discriminant validity.

Variable	*M*	SD	1	2	3	4
1 OSI	69.53	20.02	**0.822**			
2 BPNF	40.00	11.22	0.410***	**0.817**		
3 CAS	15.95	5.87	−0.418***	−0.369***	**0.821**	
4 AB	49.68	13.88	0.451***	0.385***	−0.386***	**0.811**

Bold values on the diagonal are the square roots of AVE; values below the diagonal are Pearson correlations among the constructs. *N* = 513. OSI = organizational stressors; BPNF, basic psychological need frustration; CAS, coach autonomy support; AB, athlete burnout score after reverse coding.

***p < 0.001.

**FIGURE 1:**
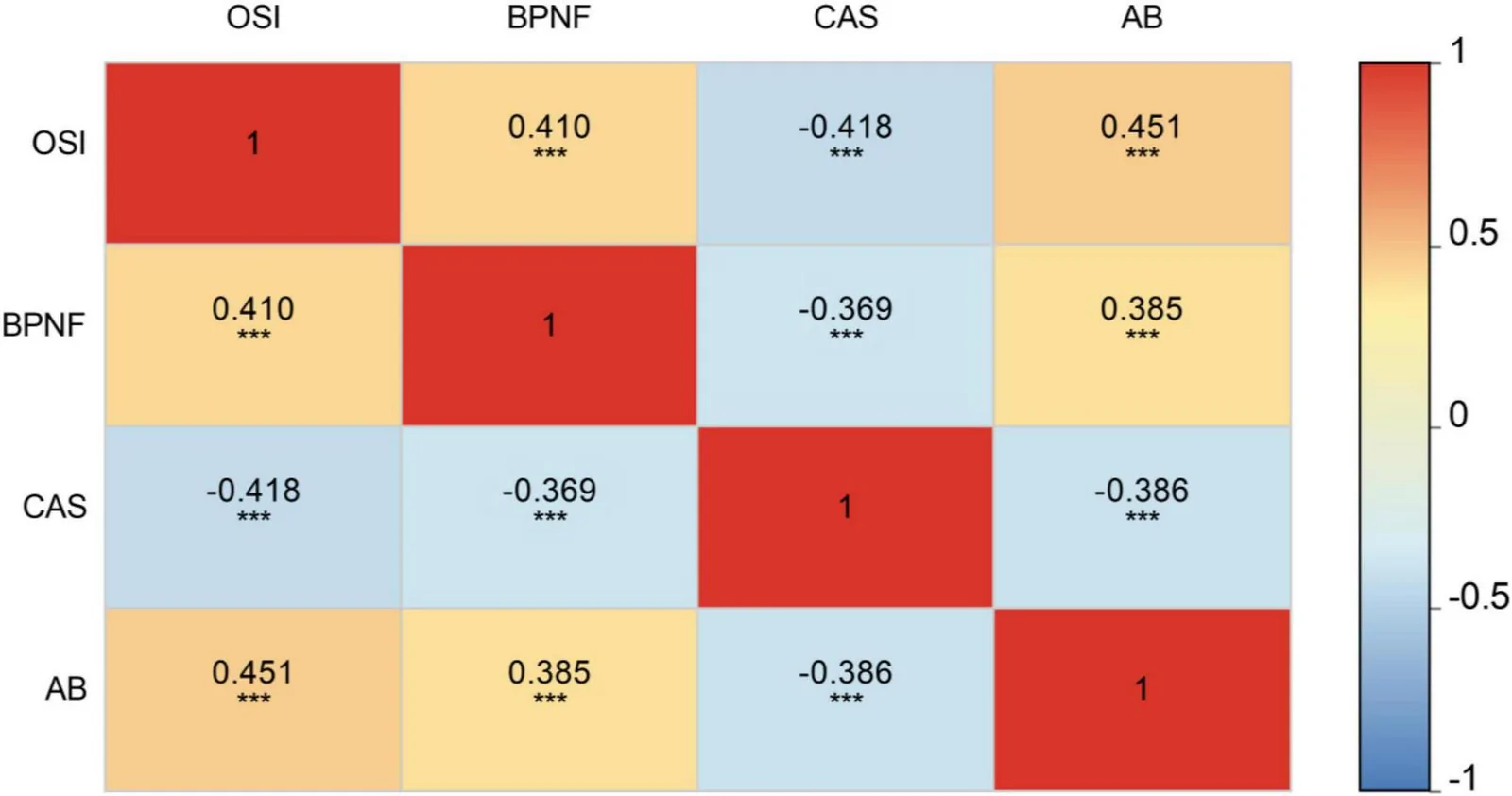
Correlation heatmap of the study variables. ***p < 0.001.

### 4.4 Direct and mediation effects

As shown in Table 5, in the baseline model without covariates (Model 0), organizational stressors (OSI) were significantly and positively associated with athlete burnout (AB; β = 0.451, *p* < 0.001), supporting H1. After adjusting for covariates including sex, age, athletic level, team role, years of training, weekly training frequency, and duration of each training session (Model 1), the positive association between OSI and AB remained significant (β = 0.446, *p* < 0.001), and the explained variance increased to 24.7%. This indicates that the association remained stable after controlling for relevant background variables.

**TABLE 5:** Path estimates for the mediation model.

Model	Path	*B*	SE	*t*	*p*	β	*R* ^2^	95% CI
Model 0	OSI → AB (c)	0.313	0.025	12.442	< 0.001	0.451	0.204	[0.263, 0.362]
	OSI → BPNF (a)	0.161	0.025	6.549	< 0.001	0.287	0.226	[0.113, 0.209]
	BPNF → AB (b)	0.297	0.051	5.837	< 0.001	0.240	0.252	[0.197, 0.398]
	OSI → AB (c’)	0.244	0.028	8.724	< 0.001	0.353	0.252	[0.189, 0.299]
Model 1	OSI → AB (c)	0.309	0.027	11.590	< 0.001	0.446	0.247	[0.257, 0.362]
	OSI → BPNF (a)	0.150	0.026	5.844	< 0.001	0.268	0.254	[0.100, 0.201]
	BPNF → AB (b)	0.301	0.052	5.748	< 0.001	0.243	0.295	[0.198, 0.404]
	OSI → AB (c’)	0.242	0.029	8.413	< 0.001	0.349	0.295	[0.185, 0.298]

Model 0 was unadjusted; Model 1 was covariate-adjusted. *a* = OSI → BPNF at mean CAS; *b* = BPNF → AB controlling for OSI; *c* = total OSI–AB association; *c*’ = direct OSI–AB association controlling for BPNF. *B* = unstandardized coefficient; β = standardized coefficient.

Further analyses showed that the positive association between OSI and basic psychological need frustration (BPNF) was significant in both the unadjusted model (β = 0.287, 95% CI [0.113, 0.209]) and the adjusted model (β = 0.268, 95% CI [0.100, 0.201]), supporting H2. When both OSI and BPNF were entered into the model, BPNF remained significantly and positively associated with AB (β = 0.243, *p* < 0.001), supporting H3. Meanwhile, after controlling for BPNF, the direct association between OSI and AB remained significant (β = 0.349, *p* < 0.001), suggesting that BPNF may play a partial mediating role between OSI and AB. Overall, the core path associations remained consistent before and after covariate adjustment, indicating that the statistical associations among OSI, BPNF, and AB were relatively robust.

The indirect association was tested using 5,000 bootstrap resamples. As shown in Table 6, the indirect association through basic psychological need frustration was significant in the unadjusted model (*B* = 0.048, Boot 95% CI [0.029, 0.069]), the main covariate-adjusted model (*B* = 0.045, Boot 95% CI [0.027, 0.067]), and the extended covariate-adjusted model (*B* = 0.046, Boot 95% CI [0.027, 0.066]). Because the bootstrap confidence intervals did not include zero in all three models, H4 was supported. These results indicate that basic psychological need frustration statistically accounted for part of the association between organizational stressors and athlete burnout, and that this indirect association remained stable after covariate adjustment.

**TABLE 6:** Bootstrap test of the mediation effect.

Model	Indirect effect	Bootstrap SE	95% Bootstrap CI
Model 0	0.048	0.01	[0.029, 0.069]
Model 1	0.045	0.01	[0.027, 0.067]
Model 2	0.046	0.01	[0.027, 0.066]

Model 1 controlled for sex, age category, athletic level, team role, years of training, weekly training frequency, and duration of each training session. Model 2 further controlled for academic year and playing position, and used ordinal trend coding for age categories. All bootstrap analyses were based on 5,000 resamples.

### 4.5 Moderating effect of coach autonomy support

The moderation analysis results are presented in Table 7. The results showed that OSI was significantly and positively associated with BPNF (*B* = 0.150, SE = 0.026, *t* = 5.844, *p* < 0.001, β = 0.268, 95% CI [0.100, 0.201]), whereas CAS was significantly and negatively associated with BPNF (*B* = −0.462, SE = 0.086, *t* = −5.390, *p* < 0.001, β = −0.242, 95% CI [−0.630, −0.294]).

**TABLE 7:** Moderating effect of coach autonomy support.

Path	*B*	SE	*t*	*p*	β	95% CI
OSI → BPNF	0.15	0.026	5.844	< 0.001	0.268	[0.100, 0.201]
CAS → BPNF	−0.462	0.086	−5.390	< 0.001	−0.242	[−0.630, −0.294]
OSI × CAS → BPNF	−0.0115	0.0043	−2.688	0.007	−0.110	[−0.0199, −0.0031]

The results are from the main covariate-adjusted model (Model 1). *R*^2^ = 0.244 before adding the interaction term, *R*^2^ = 0.254 after adding the interaction term, and Δ*R*^2^ = 0.011.

More importantly, the interaction term between OSI and CAS significantly predicted BPNF (*B* = −0.0115, SE = 0.0043, *t* = −2.688, *p* = 0.007, β = −0.110, 95% CI [−0.0199, −0.0031]). This indicates that CAS significantly moderated the statistical association between OSI and BPNF. Specifically, higher levels of CAS weakened the positive association between OSI and BPNF, suggesting that the association between organizational stressors and basic psychological need frustration was relatively weaker when CAS was higher. Therefore, H5 was supported.

To further interpret the interaction effect, simple slope analyses were conducted at low, mean, and high levels of CAS. As shown in Table 8, when CAS was low (M – 1 SD), the association between OSI and BPNF was strongest, *B* = 0.218, SE = 0.032, *t* = 6.823, *p* < 0.001, 95% CI [0.155, 0.280]. When CAS was at the mean level, the association was *B* = 0.150, SE = 0.026, *t* = 5.844, *p* < 0.001, 95% CI [0.100, 0.201]. When CAS was high (M + 1 SD), the association weakened to *B* = 0.083, SE = 0.040, *t* = 2.095, *p* = 0.037, 95% CI [0.005, 0.161]. These results indicate that the positive association between OSI and BPNF gradually weakened as CAS increased, although the magnitude of this moderation effect was small.

**TABLE 8:** Simple slope analysis.

CAS level	CAS score	Slope	SE	*t*	*p*	95% CI
Low CAS (M – 1 SD)	10.08	0.218	0.032	6.823	< 0.001	[0.155, 0.280]
Mean CAS (M)	15.947	0.15	0.026	5.844	< 0.001	[0.100, 0.201]
High CAS (M + 1 SD)	21.815	0.083	0.04	2.095	0.037	[0.005, 0.161]

The Johnson–Neyman analysis showed that coach autonomy support significantly moderated the effect of OSI on BPNF (*B* = −0.0115, *p* = 0.007, *N* = 513, *R*^2^ = 0.254). When coach autonomy support was below the boundary value of 22.10, the conditional effect of OSI on BPNF was significantly positive and became stronger as autonomy support decreased, with the 95% HC3 confidence interval excluding zero. When coach autonomy support reached or exceeded 22.10, this effect was no longer significant.

These findings indicate that when coach autonomy support was relatively low, OSI was significantly associated with higher basic psychological need frustration, whereas this association was effectively buffered under conditions of high autonomy support (Figure 2).

**FIGURE 2:**
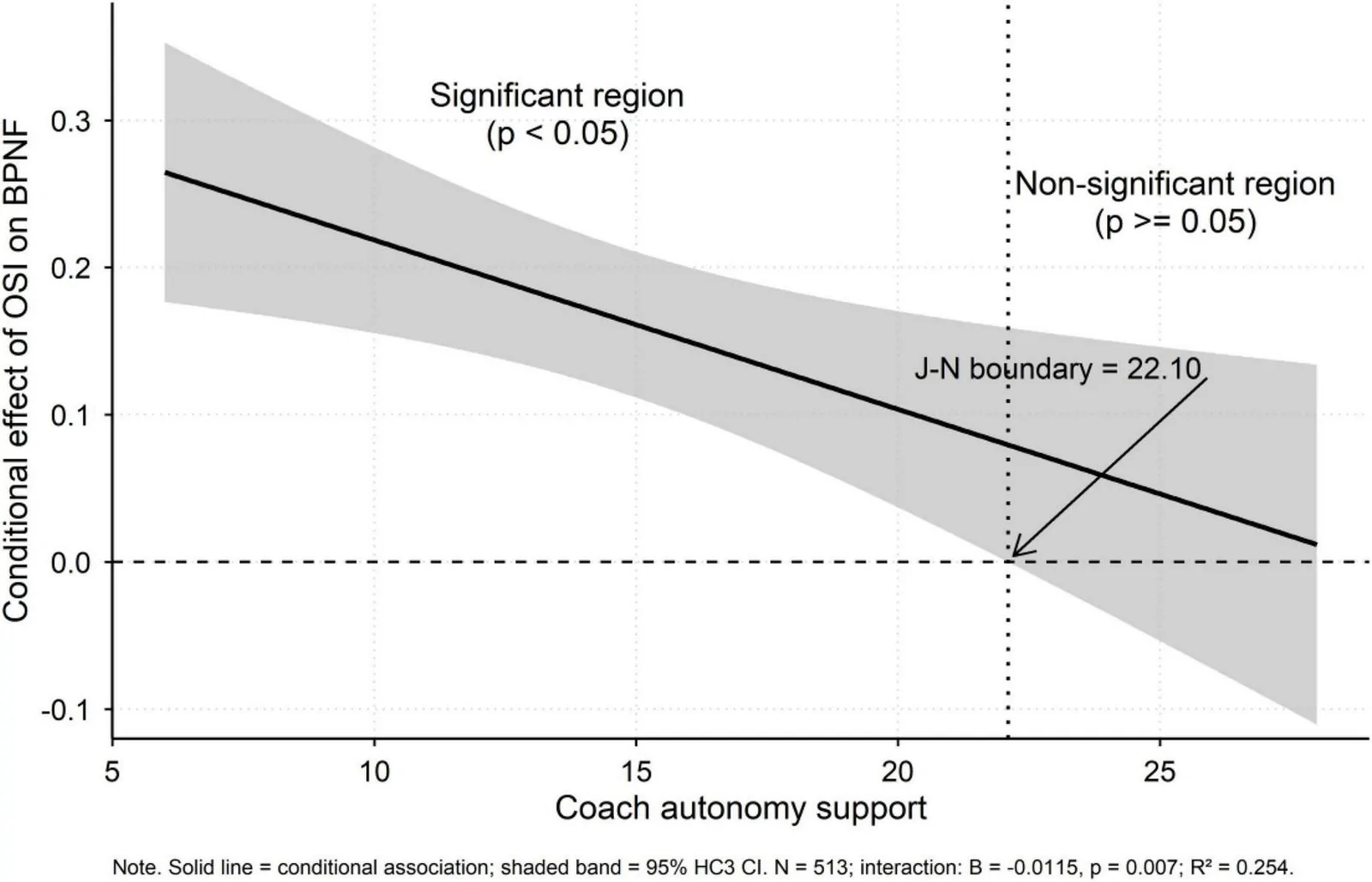
Johnson-Neyman regions of significance for the moderating effect of coach autonomy support. Solid line = conditional association; shaded band = 95% HC3 CI. *N* = 513; interaction: *B* = −0.0115, *p* = 0.007; *R*^2^ = 0.254.

### 4.6 Moderated mediation effect

As shown in Table 9, the conditional indirect effect analysis indicated that coach autonomy support (CAS) significantly moderated the indirect effect at different levels. Specifically, when CAS was low (M - 1 SD = 10.081), the indirect effect was strongest (*B* = 0.066, Boot 95% CI [0.038, 0.097]). When CAS was at the mean level (*M* = 15.947), the indirect effect was weaker (*B* = 0.045, Boot 95% CI [0.027, 0.067]). When CAS was high (M + 1 SD = 21.813), the indirect effect was further reduced but remained significant (*B* = 0.025, Boot 95% CI [0.003, 0.049]). Because the confidence intervals at all three levels did not include zero, the indirect effect was significant across all examined levels of CAS. Furthermore, the index of moderated mediation was significantly negative (*B* = −0.00346, Boot 95% CI [−0.00670, −0.00096]), and the confidence interval did not include zero. This confirms that CAS significantly moderated the mediation pathway, supporting H6.

**TABLE 9:** Conditional indirect effects and index of moderated mediation.

CAS level	CAS value	Indirect association	Boot LLCI	Boot ULCI
Low CAS (M – 1 SD)	10.081	0.066	0.038	0.097
Mean CAS (M)	15.947	0.045	0.027	0.067
High CAS (M + 1 SD)	21.813	0.025	0.003	0.049
Index of moderated mediation	–	−0.00346	−0.00670	−0.00096

As shown in Figure 3, in the main covariate-adjusted model (Model 1), OSI was significantly and positively associated with BPNF at the mean level of CAS (*B* = 0.150, β = 0.268, *p* < 0.001). BPNF was significantly and positively associated with AB after controlling for OSI (*B* = 0.301, β = 0.243, *p* < 0.001), and the direct association between OSI and AB remained significant (*B* = 0.242, β = 0.349, *p* < 0.001). The OSI × CAS interaction term was significantly negative (*B* = −0.0115, β = −0.110, *p* = 0.007), indicating that CAS negatively moderated the association between OSI and BPNF.

**FIGURE 3:**
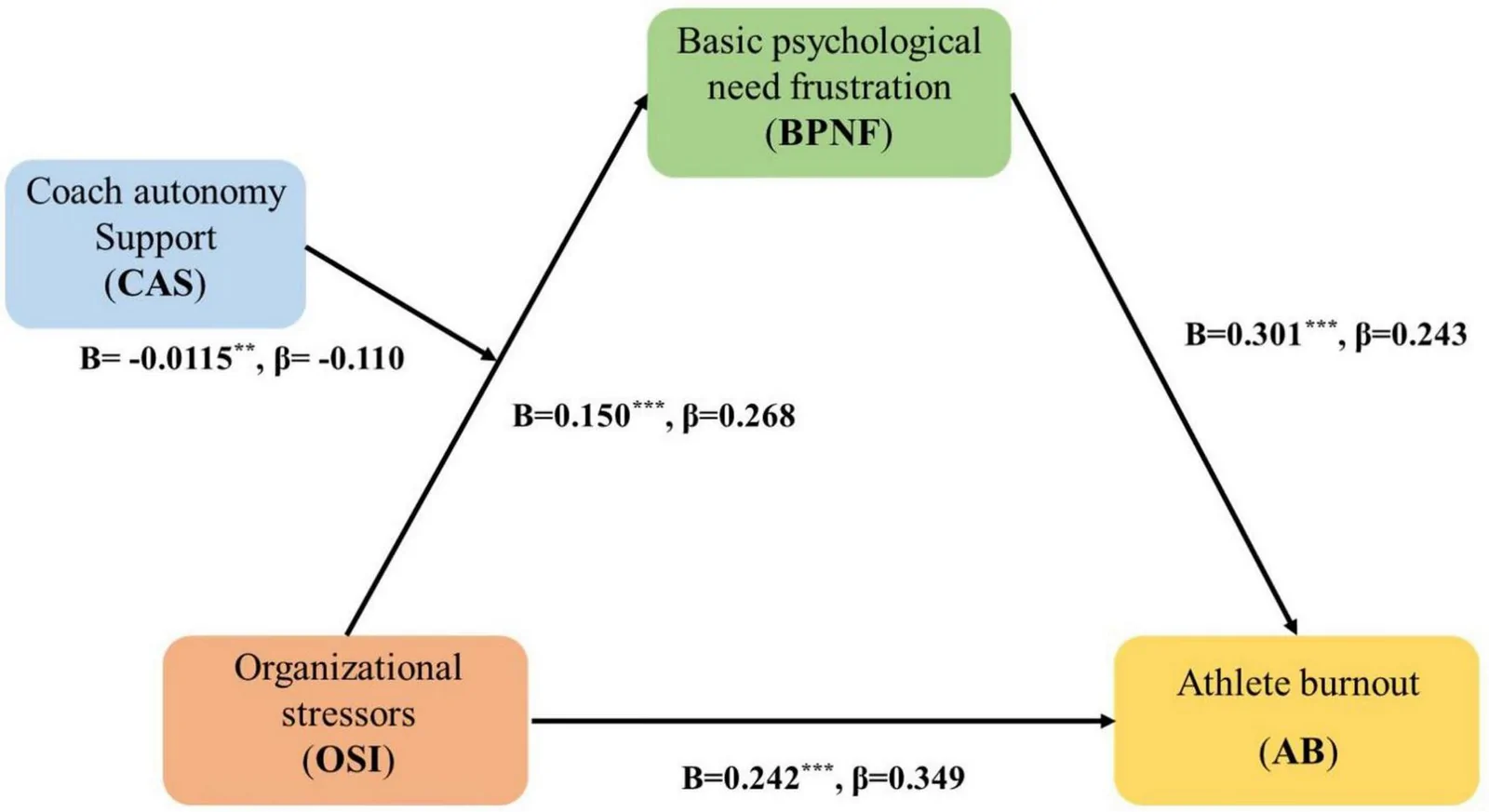
Moderated mediation model based on basic psychological need frustration and coach autonomy support. **p < 0.01; ***p < 0.001.

Together with the bootstrap results in Table 9, the index of moderated mediation was -0.00346, with a 95% bootstrap CI of [−0.00670, −0.00096]. Because the confidence interval did not include zero, the indirect association between OSI and AB through BPNF weakened as CAS increased. Therefore, H6 was supported.

### 4.7 Multicollinearity diagnostics and summary of hypothesis testing

The multicollinearity diagnostic results are presented in Table 10. In the unadjusted model (Model 0), the VIF values for mean-centered OSI, CAS, and their interaction term were 1.264, 1.236, and 1.105, respectively. After adjusting for covariates including sex, age category, athletic level, team role, years of training, weekly training frequency, and duration of each training session (Model 1), the VIF values for the three core predictors were 1.334, 1.326, and 1.138, respectively. All VIF values were well below 5, indicating that there was no serious multicollinearity among the core predictor variables.

**TABLE 10:** Multicollinearity diagnostics.

Predictor	Model 0	Model 1
OSI	1.264	1.334
CAS	1.236	1.326
OSI_x_CAS	1.105	1.138

The summary of hypothesis testing is presented in Table 11. Organizational stressors were significantly and positively associated with both athlete burnout and basic psychological need frustration. Basic psychological need frustration was significantly and positively associated with athlete burnout and statistically accounted for part of the association between organizational stressors and athlete burnout. Coach autonomy support significantly moderated the association between organizational stressors and basic psychological need frustration and further moderated the conditional indirect association between organizational stressors and athlete burnout through basic psychological need frustration. Overall, H1–H6 were supported by the statistical results.

**TABLE 11:** Summary of hypothesis testing.

Hypothesis	Decision	Corrected evidence
H1: Organizational stressors will be positively associated with athlete burnout.	Supported	Adjusted total *B* = 0.309 [0.257, 0.362]
H2: Organizational stressors will be positively associated with basic psychological need frustration.	Supported	Adjusted *B* = 0.150 [0.100, 0.201] at mean CAS
H3: Basic psychological need frustration will be positively associated with athlete burnout.	Supported	Adjusted *B* = 0.301 [0.198, 0.404]
H4: Basic psychological need frustration will account for a significant indirect association between organizational stressors and athlete burnout.	Supported	0.045 [0.027, 0.067] at mean CAS
H5: Coach autonomy support will moderate the association between organizational stressors and basic psychological need frustration, such that this association will be weaker at higher levels of coach autonomy support.	Supported	Interaction *B* = −0.0115, *p* = 0.007
H6: The indirect association between organizational stressors and athlete burnout through basic psychological need frustration will vary across levels of coach autonomy support.	Supported	Index = −0.00346 [−0.00670, −0.00096]

## 5 Discussion

### 5.1 Summary of main findings

Drawing on basic psychological needs theory, this study examined the relationships among organizational stressors, basic psychological need frustration, coach autonomy support, and athlete burnout in Chinese elite soccer players with dual educational and sport-training roles. The results showed that organizational stressors were positively associated with athlete burnout and basic psychological need frustration. Basic psychological need frustration was further positively associated with athlete burnout and statistically accounted for part of the association between organizational stressors and athlete burnout. Coach autonomy support showed a small buffering effect on the positive association between organizational stressors and basic psychological need frustration and on the conditional indirect association between organizational stressors and athlete burnout through basic psychological need frustration.

These findings suggest that burnout among elite soccer players should not be understood only in terms of training load or competitive performance pressure, but should also be examined within the organizational environment of the team. Organizational stressors, such as coach-athlete relationships, team climate, logistical management, selection procedures, and development opportunities, may represent important contextual sources of psychological strain. When these organizational demands persist without clear explanation, stable support, or effective communication, athletes may be more likely to report higher levels of burnout.

The indirect association through basic psychological need frustration further indicates that the association between organizational stressors and athlete burnout is not only direct. Organizational stressors may also be indirectly associated with athlete burnout through athletes’ sense of autonomy, competence, and relational security. In other words, athletes’ experiences of pressure, ineffectiveness, and social disconnection may help explain the observed statistical association between organizational stressors and athlete burnout.

The moderating role of coach autonomy support suggests that the relationship between organizational stressors and basic psychological need frustration depends partly on how coaches interact with athletes. Higher coach autonomy support does not mean that organizational stressors disappear. Rather, it may weaken the association between organizational stressors and need frustration by helping athletes understand training demands, acknowledging their feelings, offering appropriate choices, and reducing controlling communication. Overall, the hypotheses proposed in this study were statistically supported. Given the cross-sectional and self-report design, however, these findings should be interpreted as theory-guided statistical predictions, indirect associations, and conditional associations, rather than evidence of a strict temporal causal order among the variables.

### 5.2 Theoretical implications

A key theoretical implication of this study is that it links organizational stressors with athlete burnout and interprets this association from the perspective of basic psychological need frustration. Athlete burnout consists of emotional and physical exhaustion, reduced sense of accomplishment, and sport devaluation, and it reflects a sustained maladaptive psychological state that may develop under prolonged exposure to organizational stressors and competitive demands among elite soccer players. The present findings suggest that organizational stressors are not only directly associated with athlete burnout but may also be indirectly associated with burnout through athletes’ experiences of autonomy, competence, and relatedness frustration (Gustafsson et al., 2008). This perspective provides a theory-guided correlational pathway for identifying psychological risk among elite soccer players with dual educational and sport-training roles.

This study also provides additional evidence for the applicability of basic psychological needs theory in high-performance team sport contexts. Basic psychological needs theory emphasizes that autonomy, competence, and relatedness are important conditions for psychological integration and healthy functioning (Deci and Ryan, 2000; Vansteenkiste et al., 2020). Compared with low need satisfaction, need frustration more directly captures negative experiences involving pressure, failure, and rejection, whereas low need satisfaction mainly reflects insufficient support or a lack of resources (Bartholomew et al., 2011a; Vansteenkiste and Ryan, 2013). The findings of this study suggest that, in elite soccer settings, the association between organizational demands, such as selection uncertainty, role competition, coach feedback, and team relationships, and burnout may depend not only on whether these demands are present, but also on whether athletes experience restricted autonomy, frustrated competence, and weakened relational security in these situations.

This study further examines the boundary role of coach autonomy support in the association between organizational stressors and basic psychological need frustration. Previous research has often treated autonomy support as a social resource that promotes basic psychological need satisfaction and positive psychological functioning (Mageau and Vallerand, 2003; Adie et al., 2012; Slemp et al., 2024). In contrast, the present study positioned coach autonomy support as a moderator of the association between organizational stressors and basic psychological need frustration and found that it showed a small buffering effect. It also reduced the conditional indirect association between organizational stressors and athlete burnout through basic psychological need frustration. Thus, the theoretical role of coach autonomy support is not limited to that of a general protective factor; it can also be understood as a contextual moderator in the first-stage pathway. Coaches may not be able to remove pressures related to selection, schedules, logistics, or career development, but the ways in which they explain rules, acknowledge athletes’ feelings, provide opportunities for participation, and reduce controlling communication may shape how athletes interpret organizational demands, thereby reducing the strength of the association between organizational stressors and need frustration.

### 5.3 Practical implications

The findings of this study suggest that burnout risk among elite soccer players should not be examined only in relation to training load or individual emotion regulation. The organizational environment of the team should also be considered in daily management and future intervention research. Organizational stressors, including coach-athlete relationships, team climate, selection arrangements, logistical management, and career development pressure, may jointly constitute important sources of psychological strain for athletes with dual educational and sport-training roles. Team managers may therefore establish relatively stable mechanisms for identifying organizational stress. Questionnaires, interviews, and records of daily communication can be used to monitor athletes’ perceived pressure related to rule implementation, competition for playing opportunities, resource allocation, schedule arrangements, and team relationships. In this way, organizational stress can be transformed from a vague impression of poor condition into specific management issues that can be discussed and adjusted.

The management of organizational stressors should also attend to the predictability and explainability of team procedures. In elite soccer teams, selection, rotation, positional adjustment, and training arrangements are often necessary for competitive performance. However, when clear standards and communication channels are lacking, athletes may experience these arrangements as forced compliance or unfair treatment. In practice, coaching staff and team managers can improve the transparency of key decisions without lowering competitive demands. For example, they can explain evaluation criteria and reasons for adjustment in advance when making decisions about competition eligibility, starting and rotation roles, progressive training after injury recovery, and stage-specific goal setting. Athletes should also be given opportunities to express difficulties and receive feedback.

The indirect association through basic psychological need frustration provides a more specific perspective for identifying burnout risk. When assessing athletes’ psychological risk, teams should not focus only on fatigue or willingness to train. They should also consider whether athletes chronically feel that they have to comply, doubt their own competence, or feel socially disconnected within the team. Autonomy frustration calls for better explanation, choice, and participation. Competence frustration requires timely, specific, and actionable feedback on performance improvement. Relatedness frustration requires attention to coach communication, teammate interaction, and team belonging. If the response is limited to increasing rest or reducing training load, it may relieve some direct strain but may not address the need-related experiences that help explain the association between organizational stressors and athlete burnout.

The moderating role of coach autonomy support indicates that coaches are not only assigners of training tasks but also an important contextual source through which athletes interpret organizational stressors. In daily training, coaches can explain the purpose of high-intensity demands, provide actionable directions when criticizing performance, acknowledge athletes’ stress when adjusting roles or playing opportunities, and reduce threatening, humiliating, or one-way controlling communication. Autonomy support does not mean lowering standards. Rather, it helps athletes understand demands, identify pathways for improvement, and maintain a basic sense of psychological agency under high standards.

The practical application of these findings should still be considered in light of the study design. This study used cross-sectional self-report data, and the results support statistical prediction, indirect associations, and conditional associations among the variables. They do not show that any single management strategy will necessarily reduce athlete burnout. The suggestions above should therefore be understood as management implications based on statistical associations and theoretical mechanisms, rather than causal intervention strategies that have already been verified experimentally. Future practice may use season-long tracking, pre- and post-intervention measurement, and comparisons across teams to further examine which organizational adjustments are more effective in reducing athletes’ experiences of need frustration and improving psychological health without compromising competitive goals.

### 5.4 Limitations and future research

Several limitations should be considered when interpreting the findings and designing future studies. First, this study used a cross-sectional online questionnaire design, and all core variables were measured at the same time point. Therefore, the findings can only support theory-guided statistical predictions, indirect associations, and conditional associations, and cannot establish the temporal order among the variables. Although the proposed directions among organizational stressors, basic psychological need frustration, coach autonomy support, and athlete burnout are consistent with basic psychological needs theory and previous sport psychology research, it is also possible that athletes with higher levels of burnout are more likely to perceive organizational stressors, report greater need frustration, or perceive lower coach autonomy support. Future research could use longitudinal designs, repeated measurements within a competitive season, or cross-lagged models to further examine the temporal ordering of these variables.

Second, common method bias and the use of self-report measures limit the interpretation of the findings. This study used Harman’s single-factor test, and the first factor explained 37.75% of the variance, which did not reach the commonly used threshold for serious common method bias. The measurement model and discriminant validity results also supported the distinctiveness of the four core constructs. However, Harman’s single-factor test provides only a preliminary diagnosis and cannot fully rule out the effects of same-source measurement, a shared response context, or common response tendencies. Because organizational stressors, coach autonomy support, basic psychological need frustration, and burnout were all reported by athletes, the results may still be influenced by recall bias, current mood, social desirability, or consistency in response patterns. Future studies could separate the measurement timing of key variables and combine athlete self-reports with multiple data sources, such as coach ratings, peer ratings, training attendance records, injury information, and training load data.

Third, the representativeness of the sample should be interpreted with caution. This study included 513 soccer student-athletes enrolled in the Football College of Chengdu Sport University. These participants had relatively high competitive levels and systematic soccer-specific training experience; according to the college admission requirements and self-reported athletic-level information, all had attained at least the Chinese National First-Class Athlete level before or at university entry. The sample size was sufficient for testing the proposed model and was appropriate for examining this specific group of university-based elite soccer student-athletes. However, because data were collected through an anonymous online questionnaire, no identifiable information was recorded about participants’ current or previous teams, clubs, or training units. Therefore, the sample source and sampling procedure still have limitations. The findings are most suitable for explaining elite soccer student-athletes in a university-based soccer training and education context. They should not be directly generalized to ordinary college students, recreational athletes, full-time professional players, youth academy players, athletes in other sports, or all elite soccer players. Future research could expand the sample sources and compare model differences across competitive levels, regions, male and female athletes, professional club players, collegiate elite players, and youth academy players.

Finally, this study did not include team-level identifiers and therefore could not model the nested data structure in which athletes are embedded within different teams, coaching groups, or training units. Organizational stressors, coach autonomy support, and team culture among elite soccer players are not purely individual-level variables. Members of the same team may share similar coaching styles, selection systems, logistical conditions, and team climates. Although participants had dual educational and sport-training roles, the questionnaire did not record detailed identifiable information about specific universities, clubs, teams, or training units. This protected participant confidentiality but limited the ability to compare athletes across different organizational settings. Future studies should include team-level coding while protecting anonymity and use multilevel linear models or multilevel structural equation models to examine cross-level relationships among team-level organizational stressors, team autonomy-supportive climate, and individual need frustration. Therefore, the small interaction effect observed in the present study should be interpreted cautiously, because its statistical significance may change when multilevel modeling or cluster-robust standard errors are applied in future studies. Combining longitudinal designs, multi-source measurement, more representative samples, and team-level data would allow future research to test the temporal order, possible causal pathways, and contextual boundaries of the proposed model more rigorously.

## 6 Conclusion

Based on basic psychological needs theory, this cross-sectional study examined the relationships among organizational stressors, basic psychological need frustration, coach autonomy support, and athlete burnout in Chinese elite soccer players with dual educational and sport-training roles. The findings showed that organizational stressors were significantly and positively associated with athlete burnout, and that basic psychological need frustration statistically accounted for part of this association. Coach autonomy support showed a small buffering effect on the positive association between organizational stressors and basic psychological need frustration and further reduced the conditional indirect association between organizational stressors and athlete burnout through basic psychological need frustration. These findings suggest that management of organizational stressors, athletes’ basic psychological need experiences, and coaches’ autonomy-supportive behaviors may be relevant to understanding burnout risk among elite soccer players with dual educational and sport-training roles. Given the cross-sectional and self-report nature of the data, the conclusions should be interpreted as evidence of statistical associations and theoretically guided pathways. Future research should further test these relationships using longitudinal, multi-source, and team-level data.

## Data Availability

The original contributions presented in the study are included in the article/Supplementary material, further inquiries can be directed to the corresponding author.
